# Functional Characterization of *Lobularia maritima LmTrxh2* Gene Involved in Cold Tolerance in Tobacco through Alleviation of ROS Damage to the Plasma Membrane

**DOI:** 10.3390/ijms24033030

**Published:** 2023-02-03

**Authors:** Rania Ben Saad, Walid Ben Romdhane, Narjes Baazaoui, Mohamed Taieb Bouteraa, Yosra Chouaibi, Wissem Mnif, Anis Ben Hsouna, Miroslava Kačániová

**Affiliations:** 1 Centre of Biotechnology of Sfax, Biotechnology and Plant Improvement Laboratory, University of Sfax, B.P “1177”, Sfax 3018, Tunisia; 2 Plant Production Department, College of Food and Agricultural Sciences, King Saud University, P.O. Box 2460, Riyadh 11451, Saudi Arabia; 3 Biology Department, College of Sciences and Arts Muhayil Assir, King Khalid University, Abha 61421, Saudi Arabia; 4 Department of Chemistry, Faculty of Sciences and Arts in Balgarn, University of Bisha, Bisha 61922, Saudi Arabia; 5 Department of Environmental Sciences and Nutrition, Higher Institute of Applied Sciences and Technology of Mahdia, University of Monastir, Mahdia 5100, Tunisia; 6 Faculty of Horticulture, Institute of Horticulture, Slovak University of Agriculture, Tr. A. Hlinku 2, 949 76 Nitra, Slovakia; 7 Department of Bioenergy, Food Technology and Microbiology, Institute of Food Technology and Nutrition, University of Rzeszow, 4 Zelwerowicza St, 35601 Rzeszow, Poland

**Keywords:** *LmTrxh2*, cold stress, membrane integrity, *Lobularia maritima*, reactive oxygen species

## Abstract

Cold stress is a key environmental factor affecting plant growth and development, crop productivity, and geographic distribution. Thioredoxins (Trxs) are small proteins that are ubiquitously expressed in all organisms and implicated in several cellular processes, including redox reactions. However, their role in the regulation of cold stress in the halophyte plant *Lobularia maritima* remains unknown. We recently showed that overexpression of *LmTrxh2*, which is the gene that encodes the h-type Trx protein previously isolated from *L. maritima*, led to an enhanced tolerance to salt and osmotic stress in transgenic tobacco. This study functionally characterized the *LmTrxh2* gene via its overexpression in tobacco and explored its cold tolerance mechanisms. Results of the RT-qPCR and western blot analyses indicated differential temporal and spatial regulation of *LmTrxh2* in *L. maritima* under cold stress at 4 °C. *LmTrxh2* overexpression enhanced the cold tolerance of transgenic tobacco, as evidenced by increased germination rate, fresh weight and catalase (CAT), superoxide dismutase (SOD) and peroxidase (POD) activities; reduced malondialdehyde levels, membrane leakage, superoxide anion (O_2_^−^), and hydrogen peroxide (H_2_O_2_) levels; and higher retention of chlorophyll than in non-transgenic plants (NT). Furthermore, the transcript levels of reactive oxygen species (ROS)-related genes (*NtSOD* and *NtCAT1*), stress-responsive late embryogenis abundant protein 5 (*NtLEA5*), early response to dehydration 10C (*NtERD10C*), DRE-binding proteins 1A (*NtDREB1A*), and cold-responsive (*COR*) genes (*NtCOR15A*, *NtCOR47*, and *NtKIN1*) were upregulated in transgenic lines compared with those in NT plants under cold stress, indicating that *LmTrxh2* conferred cold stress tolerance by enhancing the ROS scavenging ability of plants, thus enabling them to maintain membrane integrity. These results suggest that *LmTrxh2* promotes cold tolerance in tobacco and provide new insight into the improvement of cold-stress resistance to cold stress in non-halophyte plants and crops.

## 1. Introduction

Plants are sessile in nature and thus face various environmental stresses throughout their life cycles, including abiotic (such as drought, heat, salinity, and cold) and biotic (such as fungi, bacteria, and viruses) factors. Among these stresses, cold causes losses in crop yields, making it a limiting factor in agricultural production [1,2]. To adapt and tolerate cold stress, plants have developed sophisticated defense mechanisms by altering biochemical and physiological processes, including the accumulation of cryoprotective polypeptides (e.g., COR15a) and osmolytes (e.g., soluble sugars and proline) [1,3,4]. Under cold stress conditions, plants generate excessive reactive oxygen species (ROS), including the superoxide radical (O_2_^.−^), hydrogen peroxide (H_2_O_2_), and singlet oxygen (^1^O_2_) [5,6], which accelerate the peroxidation of membrane lipids and even lead to cell death when they are not timely scavenged [7]. Therefore, ROS homeostasis is critical for the normal growth and development of plants. However, the most stable form of ROS, hydrogen peroxide (H_2_O_2_), diffuses freely in plants and plays a crucial role as a signaling molecule in various physiological processes [8]. Nevertheless, to regulate ROS concentrations, plant cells are also equipped with several antioxidant enzymes and proteins in various subcellular organelles, such as ascorbate peroxidase, catalase, glutathione peroxidase, peroxiredoxin (Prx), glutaredoxin, and thioredoxin (Trx) [9,10]. Numerous studies have indicated that TFs have significant roles in regulating various biological processes to protect plant cells against cold induced damage [4,11]. *NtbHLH123* expression was induced by cold stress, suggesting that *NtbHLH123* may have a regulatory role during cold stress [12]. Several studies have shown that the overexpression of *ICE1*, which results in insensitivity to chilling and freezing stresses, positively regulates *CBF* gene expression under cold stress [13]. In addition, *CBF* genes are induced by cold, and their overexpression enhances cold tolerance in transgenic *Arabidopsis*, rice, tobacco, tomato, and apple [14,15,16].

Thioredoxins represent a separate group of low molecular weight redox proteins that are controlled by a family multigene in plants [17]. Trxs have several crucial roles in many cellular processes in plants, such as the germination of seeds, the assimilation of carbon, the metabolism of lipids, phytohormone and redox signaling, and the stress response [18,19,20,21,22,23,24]. In plants, Trxs are implicated in the control of transcription factors, activate ribonucleotide reductase, enhance photosynthetic efficiency, and regulate enzyme activities [25]. Trxs are implicated in oxidative stress responses in plants by (1) repairing oxidative proteins (such as iron–sulfur protein and DNA damage repair related proteins) [21]; (2) activating the activity of protecting enzymes in the antioxidant system [26]; and (3) acting as regulators of scavenging mechanisms or signaling pathways in the antioxidant network [23,27]. In addition, the involvement of these same proteins in cold stress tolerance in rice [28] and potatoes [29] was also reported. Park et al. [30] proved that the cytosolic Trx-h2 protein from *Arabidopsis* relays the external cold stress signal to downstream cold defense signaling cascades through its protein disulfide reductase function. Lee et al. [31] showed that *Arabidopsis* disulfide reductase, Trx-h2, plays the role of a RNA chaperone under cold stress, which boosts the plant’s tolerance. Additionally, Lee et al. [32] proved that *Arabidopsis* Trx-hs regulates target proteins’ redox status via exchanging disulfide bonds between thioredoxin and the targets, especially Trx-h2, which regulates the redox status and induces the structural switching of C-repeat binding factors (CBFs). Recently, Kopeć et al. [33] reported that light plays a significant role in acquiring cold/freezing tolerance, apart from low temperatures. A low/freezing temperature disrupts the balance between light-harvesting and the subsequent processing of this energy through dark reaction biochemistry, which is downregulated under cold. Thus, the unused excess excitation energy can lead to the formation of reactive oxygen species which may interact with thioredoxins, among others. Wu et al. [34] reported that *MaTrx12* from *Musa acuminata* plays an important role in the chilling tolerance of harvested banana fruit, possibly by regulating redox homeostasis. *Trx-h1* and *Trx-h4* from *Arabidopsis thaliana* are involved in cell cycle control and proliferation [35]. *Trx-h3* is known to play a crucial role in the plant’s defense response against oxidative stress [36,37]. *Trx-h5* is important for the plant’s immune response and the structural and functional regulation of the nonexpressor of pathogenesis-related genes 1 (NPR1) [38,39]. Various studies have shown that plant *Trx-hs* genes respond to various external stresses [40,41,42,43,44]; however, the responses are species-dependent. The specific function of several Trx-h isoforms remains unclear, especially the implication of Trx-h2 in response to cold stress in the halophyte plant *L. maritima*. Based on the hypothesis that halophytes could be a valuable source of stress-adaptive genes, we previously reported that the *AlSAP* gene and its promoter isolated from the halpoyte plant *Aeluropus littoralis* were induced by high- and low-temperature stress [45,46]. The constitutive expression of *AlSAP* in tobacco and rice has resulted in an enhanced tolerance to cold, heat, drought, salinity, and oxidative stresses [45,47]. Recently, *AlTMP1* gene encoding the plasma membrane protein 1 from *A. littoralis* was found to be inducible by low-temperature (4 °C), and its overexpression in transgenic tobacco conferred tolerance to freezing (−20 °C for 2 h) and heat (55 °C for 2 h) stresses [48]. Previously, we isolated and characterized the first *Trxh2* gene of the halotolerant plant *L. maritima*, which was designated as *LmTrxh2* and deposited in the GenBank databases (accession number: MW589650) [49]. LmTrxh2 is a thioredoxin H-type protein characterized by the presence of a conserved TRX domain and catalytic Trx motif CGPC, which exhibits 91.1% similarity with *Arabidopsis thaliana* [49]. The *LmTrxh2* gene is inducible by salt and osmotic and oxidative stresses in *L. maritima* localized in the plasma membrane, and its overexpression in transgenic tobacco plants resulted in an enhanced tolerance to salt and osmotic stresses [49]. The aim of this work was therefore to study the role of *LmTrxh2* under cold stress. We functionally characterized a differentially expressed *LmTrxh2* gene in *L. maritima* via inducible expression pattern analysis. *LmTrxh2*-overexpressed tobacco plants were investigated in cold stress conditions, and the experimental results showed that an overexpression of *LmTrxh2* in tobacco plants conferred them tolerance to cold stress. Furthermore, these results might help in clarifying the response mechanisms of *LmTrxh2* under cold stress condition.

## 2. Results

### 2.1. LmTrhx2 Transcription Is Highly Induced by Cold Stress

**Figure 1:**
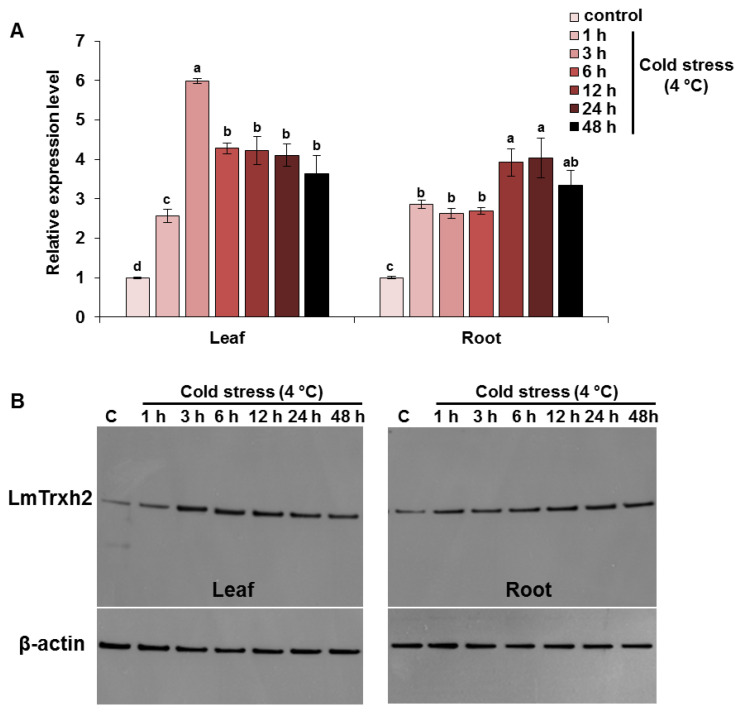
*LmTrxh2* expression profiles and protein accumulation in response to cold stress (4 °C). (**A**) Pattern of expression of the *LmTrxh2* gene and (**B**) the production of LmTrxh2 protein in the leaves and roots of *L. maritima* plants following the application of cold stress for 48 h. A western blot analysis of the total protein extracts (10 µg) was performed using anti-LmTrxh2 rabbit IgG (H + L) antibodies on leaves and roots tissues. The upper panel shows the detection of LmTrxh2, and the lower panel shows anti-β-actin for loading control. Vertical bars indicate the standard deviation calculated from three replicates. Values are mean ± SEM (*n* = 3). Different lowercases indicate a significant difference at *p* < 0.05.

To identify the cold stress response of *LmTrhx2*, we exposed the *L. maritima* seedlings to cold stress and used RT-qPCR to analyze the relative expression level of *LmTrhx2*. When stressed at 4 °C, we observed that the *LmTrhx2* mRNA level significantly increased in the leaves, with a 2.5-fold expression at 1 h, while it reached a peak (6-fold) at 3 h, and it decreased slightly at 24 h (Figure 1A). *LmTrhx2* was also remarkably induced in the root within 1 h of the treatment and reached a peak at 12 h (4-fold induction) (Figure 1A). A western blot analysis technique was conducted to determine whether the transcription level of *LmTrxh2* was correlated with the accumulation of protein levels in leaves and roots tissues that were treated or not treated with cold stress. Indeed, the immunoblots analysis confirmed the above obtained results of *LmTrxh2* expression and suggested that the LmTrxh2 protein level was abundant in the leaves and roots of *L. maritima* under cold stress (Figure 1B). Hence, these results suggest that cold stress induces *LmTrxh2* transcription and protein accumulation in *L. maritima*.

### 2.2. Transgenic Tobacco Plants Harboring LmTrxh2 Gene Enhanced Cold Tolerance

We have used three previously established homozygous *LmTrxh2* tobacco lines (Tr1, Tr2, and Tr3) that constitutively express *LmTrxh2* gene, as reported previously [49]. Then, their tolerance responses to cold stress (4 °C) were investigated. Seeds of Tr1, Tr2, Tr3 and NT lines were sown in vitro on MS media under 25 °C or 4 °C temperature, and the germination rates were determined afterwards. In fact, transgenic and NT seeds had the same germination levels under 25 °C, and all seeds had germinated after 10 days.

**Figure 2:**
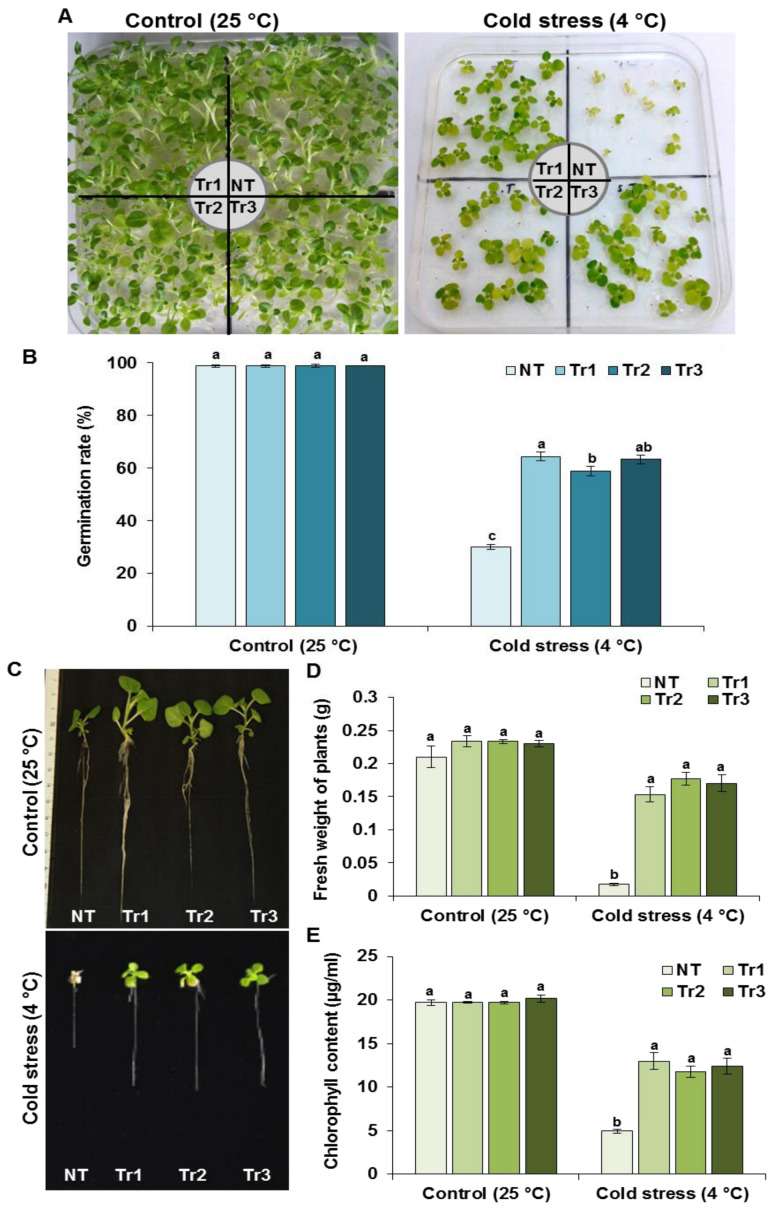
Assessment of NT and *LmTrxh2* transgenic lines performance at germination stage under cold stress conditions. (**A**) Photographs were taken two weeks after seed germination. (**B**) Seed germination rates were determined for *LmTrxh2* overexpressors lines and NT plants under normal (25 °C) and cold stress (4 °C) conditions. The results presented are the means of three independent biological replicates, and a minimum of 30 seeds were counted for each experiment. (**C**) Comparison of the growth of transgenic and NT plants on plates. Tobacco seedlings were grown vertically for two weeks, and the fresh weight (**D**) and total chlorophyll content were measured (**E**) under normal and cold stress conditions. Data are expressed as the mean ± SEM (*n* = 3). Different lowercases indicate a significant difference at *p* < 0.05.

As illustrated in Figure 2A, the three transgenic lines were phenotypically characterized by more vigorous growth and greener leaves and significantly higher germination rates compared with NT plants under cold stress (4 °C). Indeed, 58–64% of seeds from the previously reported three transgenic lines had germinated compared to only 30% of the NT seeds. Therefore, this indicates that *Lmtrxh2* overexpression in tobacco plants may have enhanced the seeds’ germination tolerance to cold stress (Figure 2B). As shown in Figure 2C, transgenic lines showed better growth parameters compared with the NT plants. In fact, fresh weight accumulation, shoot length, and root length were significantly higher in the transgenic lines (Tr1, Tr2, and Tr3) than those in the NT plants (Figure 2D and Figure S1). We further measured the total chlorophyll content in the three transgenic lines and found that following cold treatment, the total chlorophyll levels of Tr1 (12.97 µg/mL), Tr2 (11.50 µg/mL), and Tr3 (12.38 µg/mL) transgenic plants were higher than the NT plants (4.96 µg/mL) (Figure 2E).

### 2.3. LmTrxh2 Overexpression Alleviated Free Radical Damage on the Membrane under Cold Stress

**Figure 3:**
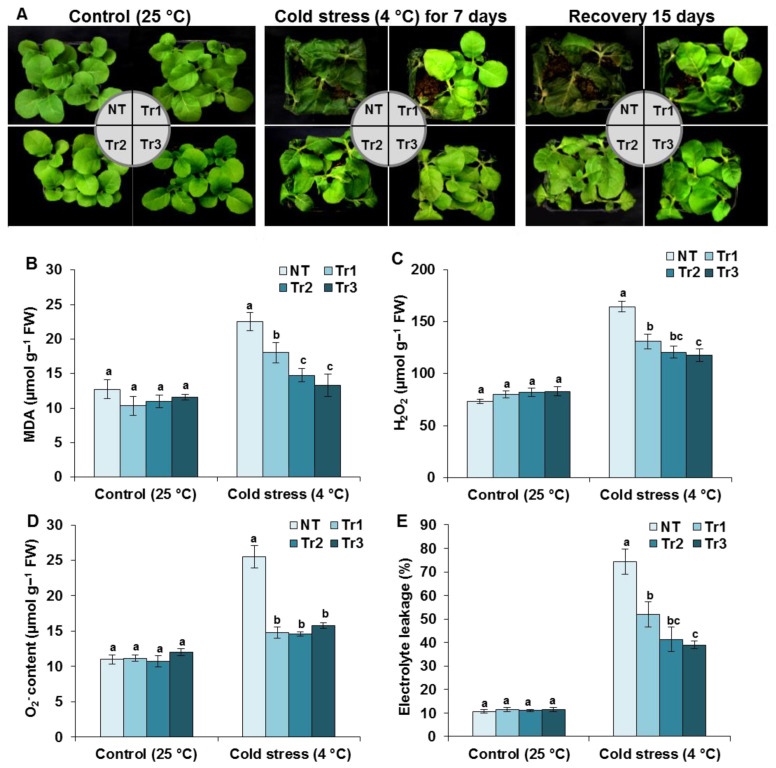
The *LmTrxh2* overexpressing plant had increased cold tolerance. (**A**) The phenotypic appearance of 45-day old seedlings for NT and transgenic plants following the application of cold stress (4 °C) for 7 days, followed by recovery at 25 °C for 15 days. (**B**) Quantification of MDA, (**C**) H_2_O_2,_ and (**D**) O_2_^−^ accumulation in the leaves of NT and transgenic tobacco lines under control or subjected to cold stress (4 °C for 48 h). (**E**) The electrolyte leakage analysis of NT and *LmTrxh2* transgenic lines under normal growth conditions and after cold treatment (4 °C for 48 h). The average of three independent experiments ± SEM is shown. Different lowercases indicate a significant difference at *p* < 0.05.

Seedlings that were 45 days old were used to further investigate cold stress tolerance in tobacco plants. No morphological differences were observed between transgenic and NT plants before cold treatment (Figure 3A); however, when the seedlings were subjected to cold stress (4 °C) for 7 days, serious cold injuries were recorded in the NT plants compared with the transgenic plants. Following a 15-day recovery period under 25 °C, most of the NT plants died, while the majority of the *LmTrxh2*-overexpressing plants survived (Figure 3A).

One serious drawback of cold stress exposure is the generation of ROS, which stimulates oxidative stress and consequently results in a damaging effect on the different cell components. Furthermore, malonaldehyde (MDA) is an important indicator regarding the degree of lipid peroxidation which is commonly used to estimate membrane damage under oxidative stress [50]. Thus, we evaluated the accumulation of H_2_O_2_ and the content of lipid peroxidation in *LmTrxh2* transgenic seedlings that were grown under normal or cold stress conditions. Although there was no difference between the MDA (Figure 3B) and H_2_O_2_ (Figure 3C) contents in NT and transgenic plants under normal conditions, the transgenic plants had significantly lower MDA and H_2_O_2_ contents than the NT plants under cold stress. The rate of O_2_^−^ accumulation in the leaves of transgenic lines and NT plants was also quantified spectrophotometrically (Figure 3D). It increased after 48 h of cold stress in three transgenic lines but at significantly lower levels than the NT plants. We can conclude from this data that the overexpression of *LmTrxh2* had an inhibitory effect on the accumulation of ROS induced by cold stress in plants. We further measured the El to determine the membrane integrity of the plants under cold stress. No significant differences were found between transgenic and NT leaves under normal conditions, while El values were significantly lower in transgenic leaves under cold stress (Figure 3E).

Since the tobacco plants overexpressing *LmTrxh2* had enhanced cold tolerance, we subjected transgenic and NT leaves to NBT and DAB staining in order to detect O_2_^−^ and H_2_O_2_ production in young seedlings treated with cold stress for 48 h.

**Figure 4:**
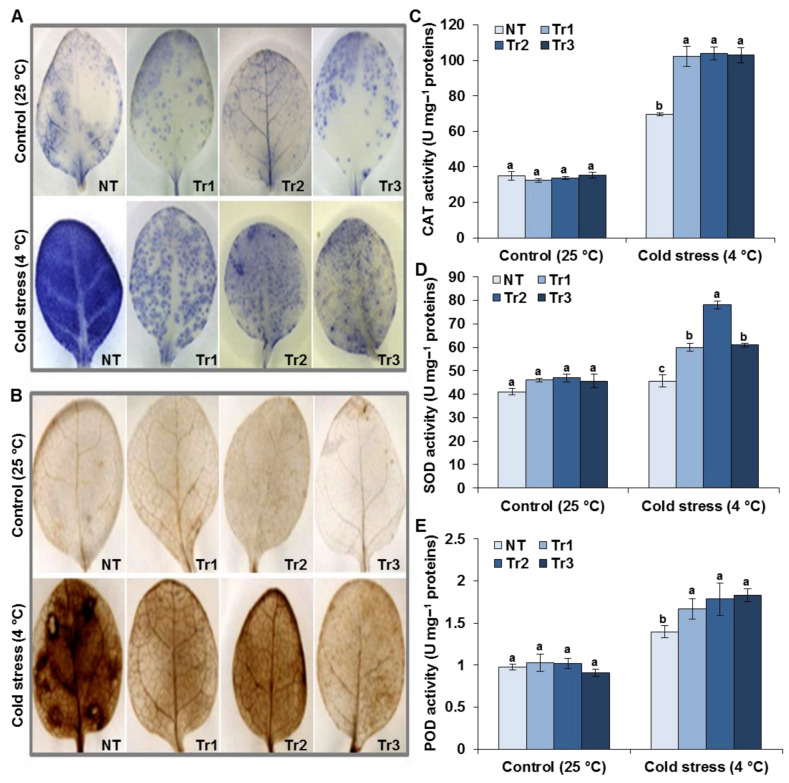
Physiological indices of NT and *LmTrxh2* transgenic plants under control and cold stress conditions. The leaves were sampled from plants grown under normal growth conditions and plants subjected to 48 h of cold stress (4 °C). (**A**) NBT and (**B**) DAB staining of leaves. (**C**) CAT activity. (**D**) SOD activity. (**E**) POD activity. Values are presented as means ± SEM values (*n* = 3). Different letters indicate a significant difference at *p* < 0.05.

As shown in Figure 4A,B, under normal conditions, no significant differences were found in the accumulation of O_2_^−^ and H_2_O_2_ between the leaves of *LmTrxh2* transgenic tobacco and those of the NT plants. However, under cold treatment, the NBT and DAB staining were stronger and intensified in all lines. Nevertheless, in the *LmTrxh2*-overexpressing lines, exposure to cold stress led to less accumulation of O_2_^−^ and H_2_O_2_ compared with the NT plants.

Our results thus suggest that the accumulation of ROS was higher in the NT plants compared with the transgenic plants, and this could be due to the activation of three key antioxidant enzymes (CAT, POD, and SOD), which are crucial in ROS scavenging and ROS cellular homeostasis [51]. We hence quantified their levels before and after cold stress treatments and found that the levels of the expression of the three proteins were similar between the NT and transgenic plants under normal conditions. Following cold treatment, however, *LmTrxh2*-overexpressing plants showed an enhancement in the activities of all antioxidant enzymes. In contrast, the levels of these same proteins in the NT plants only slightly increased (Figure 4C–E). These data suggest that *LmTrxh2* overexpression could alleviate membrane damage by enhancing the free radical scavenging ability of tobacco, thus promoting its cold stress tolerance.

### 2.4. LmTrxh2 Altered the Expression Levels of the Cold-Responsive and ROS Scavenging-Related Genes

**Figure 5:**
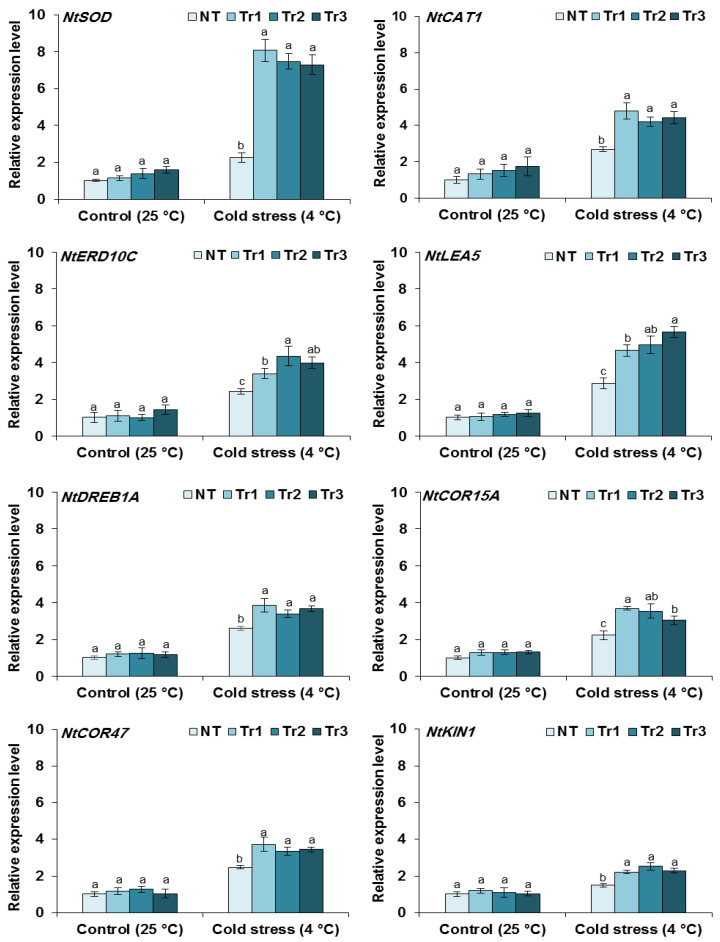
Relative expression of ROS-related (*NtSOD* and *NtCAT1*) and cold-responsive genes (*NtERD10C*, *NtLEA5*, *NtDREB1A*, *NtCOR15A*, *NtCOR47*, and *NtKIN1*) in NT and transgenic lines before and after 48 h of cold stress (4 °C). Values are presented as means ± SEM values (*n* = 3). Different letters indicate a significant difference at *p* < 0.05.

The enhanced cold tolerance in transgenic tobacco plants was conferred by the *LmTrxh2* overexpression-induced enhancement of the free radical scavenging ability. Consequently, an induction in the expression of ROS-related genes led to an enhanced production of enzymes that were implicated in direct ROS detoxification in the NT and transgenic plants before and after cold treatment. The transcript levels of *NtSOD* and *NtCAT1* were similar between NT and transgenic lines under normal conditions but were significantly upregulated in the transgenic lines compared with the NT under cold treatment. This suggests that *LmTrxh2* could be a key regulator gene that may be found upstream of some ROS-related genes. Therefore, the overexpression of *LmTrxh2* could result in the activation of the expression of several ROS-related genes that may help plants positively cope with environmental stresses. Additionally, we investigated several transcript levels of many cold stress defensive proteins (*NtDREB1A*, *NtCOR15A*, *NtCOR47*, *NtKIN1*, *NtLEA5*, and *NtERD10D*). The results showed that following cold stress, the expression levels of the cold-related genes enhanced 4.6- to 5.6-fold in *LmTrxh2* transgenic lines compared with NT plants (Figure 5). These results, therefore, suggest that the overexpression of *LmTrxh2* may increase the expression levels of the mRNA of ROS scavenging-related and stress-responsive genes following cold stress.

## 3. Discussion

In this study, the *LmTrxh2* gene from *L. maritima* was functionally demonstrated to be implicated in the regulation of the plant’s cold tolerance. This effect was attributed to an enhanced H_2_O_2_-scavenging capacity and intact membrane integrity maintenance induced by *LmTrxh2* overexpression and the upregulation of cold stress defensive genes. In a previous report, we showed that the *LmTrxh2* gene enhanced salt and osmotic stress tolerance to tobacco through the regulation of redox homeostasis [49]. In this study, our results showed that the expression of the *LmTrxh2* gene was differentially induced in the leaves and roots of *L. maritima* by a low temperature, suggesting that *LmTrxh2* may have a regulatory role during cold stress. Duan et al. [52] showed that the expression level of the *MsTRX* gene was significantly altered by different abiotic stresses, such as cold, drought, and salt in alfalfa. Furthermore, mRNA level of *Arabidopsis* NADPH-dependent thioredoxin reductase C (*AtNTRC*) was also induced in response to cold stress, which confers freezing and cold shock tolerance to plants [53]. In addition, several *Trxs* have been found to accumulate specifically in certain tissues. Similar to our results, the expressions of *LjTrxf*, *LjTrxm1*, *LjTrxm4*, and *LjTrxx* in *Lotus japonicas* were higher in leaves than in roots [54]. For instance, pea thioredoxin *PsTRXf1* was found in early seedlings, leaves, roots, stems, and flowers, while the expression of *PsTRXm1* was restricted to leaves, roots, seeds, and flowers [55]. *Trxs h2* (*MtTrx1*) and *h6* (*MtTrx31*) were abundant in cotyledons of the legume *Medicago truncatula* [56]. Similarly, Zhang et al. [23] reported that an *h-type Trx* gene in *Oryza sativa* (*OsTrxh1*) was induced by salt stress in root tissues and can influence the content of ROS by regulating the apoplastic ascorbate system or other antioxidant enzymatic activities. An upregulation of a *Glycina max* h-type *Trx* gene (*GmTrxh*) in the roots and infected cells of mature nodules after inoculation with a nodulating bacterium was observed, and this gene was found to contain an antioxidant responsive element (ARE)-like site in its 5′ upstream region, and its cDNA conferred tolerance to H_2_O_2_ in a yeast thioredoxin mutant [57]. Collectively, we conclude from these findings that the modulation of the *LmTrxh2* gene in leaves and roots might be associated with cold tolerance mechanisms in *L. maritima.*

*TRX* genes were reported to be crucial for the regulation of biological mechanisms implicated in the protection of plants against cold-induced damages [40,43]. In the present work, we showed that following the stress cold tolerance assay, the transgenic tobacco plants were more capable of tolerating cold stress compared with the NT ones. This was proven through the measurement of the survival rate, EL (%), MDA, H_2_O_2_, and total chlorophyll levels, which were in accordance with the phenotypic observation that suggested that the overexpression of *LmTrxh2* enhanced cold tolerance. Likewise, Park et al. [37] showed that the overexpression of the *Arabidopsis Trx-h3* gene conferred heat shock tolerance in plants. Additionally, in harvested banana, the gene *MaTrx12* was shown to be involved in cold tolerance [34], and the overexpression of *AtTrx-h2* gene was found to confer resistance to salt stress in *Brassica napus* [58]. Similarly, the overexpression of the gene *GhTRX134* in *Arabidopsis* was reported to enhance drought and salt tolerance as well as resistance to oxidative stress [59]. Following exposure to cold stress, the balance between ROS production and clearance is known to be disrupted [5,51], which results in multiple cytological effects that may include plasma membrane lipid peroxidation [60]. In fact, plasma membrane integrity is directly correlated with the degree of membrane lipid peroxidation. Therefore, this implies that efficient scavenging of ROS in the plasma membrane may endow plants with higher cold tolerance. Our subcellular location analysis indicated that LmTrxh2 is localized at the membrane and cytoplasm [49], suggesting its role in the degradation of over-accumulated H_2_O_2_ in the membrane under cold stress. Nevertheless, the regulation of the ROS system under different abiotic stresses remains dependent on the ROS-scavenging systems, especially through the antioxidant enzymes SOD, CAT, and POD [5,61]. These enzymes are regulated by *TRX* genes [62] and were found to be highly expressed under cold stress in the transgenic plants compared with the NT ones. Their scavenging activity was also indirectly proven by the reduction of H_2_O_2_ and MDA contents under cold stress in the transgenic plants.

In the present study, we further showed that the two ROS-scavenging related genes *NtSOD* and *NtCAT1* were highly expressed in the *LmTrxh2*-overexpressing plants compared with the NT plants under cold stress. Indeed, the level of expression of these two genes correlated with the enhanced activities of the three previously mentioned antioxidant enzymes. Therefore, we suggest that the overexpression of *LmTrxh2* could enhance cold tolerance due to a better ROS-scavenging system.

One of the regulator genes that plays a crucial role in the cold adaptation of plants is the cold-regulated (*COR*) gene. Studies have shown that about 10–20% of the *COR* gene is regulated by C-repeat binding factors (*CBFs*) [63,64,65]; for example, expressions of 24 *COR* genes are regulated by CBF-dependent cold-induced zinc finger transcription factor *ZAT12* [66]. Cold stress also induces the expression of late embryogenesis abundant (*LEA*), *COR15A*, and *COR15B* proteins, which can interact with membranes and act as membrane protectants [67]. The expression of the *COR15A* gene that encodes a hydrophilic protein has been reported to improve the cold tolerance of the chloroplasts of domestic plants [68]. In addition, *COR47* is also closely related to cold tolerance because it acts as an anti-dehydrating agent that prevents excessive dehydration of plant cells due to low temperatures [69]. Interestingly, RT-qPCR analysis before and after cold stress showed that the mRNA levels of cold-responsive genes, such as *DREB1A* (dehydration response element B1A), *COR15A* (cold-regulated 15A), COR47 (cold-regulated 47), *KIN1* (stress-responsive protein KIN1), *NtERD10D* (early response to dehydration 10D), and *NtLEA5* (late embryogenesis abundant protein) or their homologs respond to abiotic stresses [70,71]. They were found to be strongly expressed in the transgenic plants compared with the NT ones, suggesting minimal damage to those plants, which is evidenced by high membrane integrity under cold stress. In future studies, we plan to unravel whether the *LmTrxh2* gene was directly implicated in the regulation of the stress-responsive genes to improve plant cold-stress resistance.

This study functionally characterized the *LmTrxh2* gene from *L. maritima*, which specifically enhanced the tolerance of tobacco plants to cold stress. This specific response could be attributed to its role in ROS scavenging and the upregulation of stress-responsive genes to maintain plasma membrane integrity.

## 4. Materials and Methods

### 4.1. Plant Materials and Growth Conditions

Sample of seeds of *L. maritima* were taken from saline marshes near the region of Chebba, Mahdia in Tunisia. They were then dried and stored at 4 °C until further use. Sterilization and germination procedures of the seeds were done following protocols previously described by Ben Saad et al. [72]. Seedlings were then left to grow for four weeks in a nutrient solution, as reported elsewhere [73], and they were also subjected to cold treatment. The treatment consisted of exposing the plants to a low temperature (4 °C) under an illumination scheme of 16 h/8 h (light/dark) with a light intensity of 35 μmol m^−2^ s^−1^. Thereafter, plants tissues were collected at 1, 3, 6, 12, 24, and 48 h and were immediately frozen in liquid nitrogen and preserved at −80 °C for further analyses.

### 4.2. RNA Isolation, cDNA Synthesis, and Quantitative Reverse Transcription PCR Analysis

In order to analyze the expression of *LmTrxh2* transcript in *L. maritima*, the leaves and roots of *L. maritima* were collected at 1, 3, 6, 12, 24, and 48 h of cold stress (4 °C). RNA isolation and cDNA synthesis were performed according to our previously published method [49]. Briefly, RNA from *L. maritima* and transgenic tobacco plants were extracted using the standard TRIzol method. The obtained RNA was treated with DNase I (MBI Fermentas, Hanover, MD, USA) at 37 °C for 15 min to remove any remaining genomic DNA and used as a template for cDNA synthesis [74]. RT-qPCR were conducted as described by Ben Saad et al. [74]. The forward and reverse primers (qLmTrxh2-F and qLmTrxh2-R, respectively) were used for the RT-qPCR analysis of *LmTrxh2* expression. The *UBQ10* gene (UBQ10-F and UBQ10-R) was used as an internal control, and the relative expression of the target was calculated using the 2^−ΔΔCT^ method [75]. Three technical replicates and three biological replicates were used for each treatment. The primer sequences used for the RT-qPCR are listed in Supplementary Table S1. The RT-qPCR was also used to determine the transcript accumulation of *LmTrxh2* in homozygous transgenic tobacco lines (Tr1, Tr2, and Tr3) and NT tobacco plants and to monitor the expression levels of ROS-related (*NtSOD* and *NtCAT1*) and cold-response genes (*NtDREB1A*, *NtCOR15A*, *NtCOR47*, *NtKIN1*, *NtLEA5* and *NtERD10C*) (Table S1) in Tr1, Tr2, and Tr3 transgenic lines exposed to 4 °C for 48 h. Total RNA isolation from tobacco leaf tissues, cDNA synthesis, and RT-qPCR using gene specific primers were performed as described above. The *Actin* gene (ACT-F and ACT-R) was used as an internal control (Table S1).

### 4.3. Western Blot Analysis

The Western blotting method was used to quantify the accumulation of *LmTrxh2* in the tissues of the leaves and roots of *L. maritima* that were collected after 1, 3, 6, 12, 24, and 48 h of cold treatment (4 °C). Total proteins extraction, separation, and LmTrxh2 protein detection were conducted as previously described by Ben Saad et al. [76]. Monoclonal antibody to β-actin was purchased from Sigma Aldrich Co., St. Louis, MO, USA (Cat#A5441).

### 4.4. Cold Tolerance Analysis of the Transgenic Plants

The seeds of transgenic homozygous T3 generation tobacco lines (Tr1, Tr2, and Tr3) and NT plants were surface-disinfected and germinated on MS medium and incubated for 7 days at 25 ± 1 °C or 4 ± 1 °C. Their germination rates were recorded, and the images were captured two weeks after growth in a culture chamber under a 16 h/8 h light/dark cycle at 25 °C. After a two-week growth period, the fresh weight of the plants and the chlorophyll content of the leaves were determined. The total chlorophyll content in each sample was calculated after extraction in aqueous 80% acetone [77] using the following formulae which express [Chl a], [Chl b], and [Chls a + b] in µg/mL: [Chl a] = 12.70 × A663 − 2.69 × A645, [Chl b] = 22.90 × A645 − 4.68 × A663, and [Chls a + b] = 20.21 × A645 + 8.02 × A663. The A663 and A645 represent absorbance values read at 663 nm and 645 nm wavelengths, respectively. The assays were conducted in triplicate on independent seed lots.

Regarding the analysis of the grown seedlings, the plants were first put into plastic containers that were filled with both soil and sand (1:1) under controlled conditions (25 °C temperature, 16 h light/8 h dark, 70% humidity, and 200 µM m^−2^ s^−1^ light intensity). Then, the transgenic and NT transgenic tobacco plants were aged of 45 days. The 45-day-old tobacco plants of the NT and transgenic plants (Tr1, Tr2, and Tr3) were transferred to the growth chamber under the previously mentioned controlled conditions for 7 days. Thereafter, the cold-stressed plants were returned to the normal growth chamber to grow for 15 more days. Then, tissues of the treated plants (4 °C for 48 h) and the control plants were collected for biochemical (oxidative stress markers and antioxidant enzyme activities) analysis and electrolyte leakage (El). All the results were based on the average of three independent biological replicates.

### 4.5. Histochemical Staining Analysis

The hydrogen peroxide (H_2_O_2_) and superoxide radicals (O_2_^−^) accumulation in the leaves were stained using DAB and NBT solution, as described by Ben Hsouna et al. [78]. Briefly, leaves that were stained with DAB or NBT were treated with 70% ethanol to remove the chlorophyll content and make the staining more visible. Then, the stained slides were observed and photographed with a Leica MZ FLIII binocular microscope (Leica Microsystems, Heerbrugg, Switzerland).

### 4.6. Determination of the Physiological Indexes

The activities of antioxidant enzymes superoxide dismutase (SOD), peroxidase (POD), catalase (CAT), and the content of malondialdehyde (MDA) and H_2_O_2_ were measured as previously described [79]. Superoxide content (O_2_^−^) was quantitated as described by Chen et al. [80]. Furthermore, the El was measured following a previously published method [48]. Furthermore, the total chlorophyll content of the leaves was determined as described previously by Ben Romdhane et al. [72].

### 4.7. Statistical Analysis

Data were analyzed using the Statistical Analysis System software (IBM SPSS Statistics 21.0, IBM Corp., Armonk, NY, USA), and significant differences among treatments were determined using Duncan’s multiple range test (*p* < 0.05). The data were presented as means ± SD values of three biological replicates (*n* = 3).

## Data Availability

All data is contained within the article or supplementary material.

## Supplementary Materials

The supporting information can be downloaded at: https://www.mdpi.com/article/10.3390/ijms24033030/s1.
