# Supplementary material for: Functional Characterization of Lobularia maritima LmTrxh2 Gene Involved in Cold Tolerance in Tobacco through Alleviation of ROS Damage to the Plasma Membrane
Source: Int J Mol Sci. 2023 Feb 3;24(3):3030. doi: 10.3390/ijms24033030 (PMC9917683; doi:10.3390/ijms24033030)
Supplement: Supplementary file 1 [file ijms-24-03030-s001.zip › ijms-2121744-supplementary.pdf]

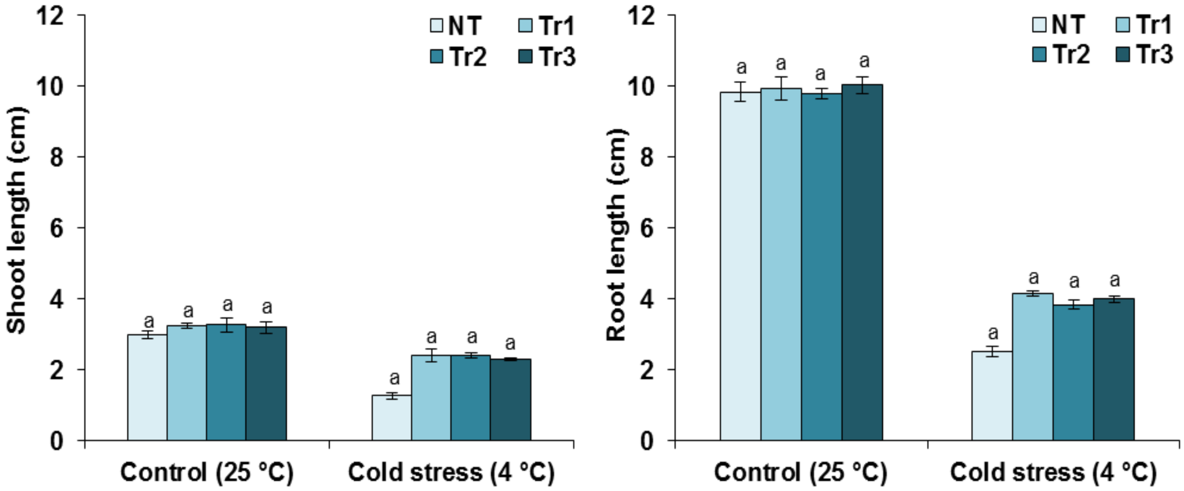

Figure S1. Effect of cold stress on shoot and root elongation of NT and *LmTrxh2*-transgenic tobacco seedlings. Data are expressed as the mean ± SEM (n =3). Different lowercases indicate significant difference at P< 0.05.

Table S1. Sequences of primers used in PCR and RT-qPCR analysis.

Primers	Sequences
**q*LmTrxh2*-F**	5′-TGCTGGTGGTGGATTTTAGC-3′
**q*LmTrxh2*-R**	5′-CAAAATCCGGCAGTTCATC-3′
**q*UBQ-10* -F**	5′-GAATACCTCCTTGTCCTGGATCT-3′
**q*UBQ-10*-R**	5′-GTACTTTGGCGGATTACAACATC-3′
**q*ACT*-F**	5'- GTGCCCATTTACGAACGATA-3'
**q*ACT*-R**	5'-GAAGACTCCATGCCGATCAT-3'
**q*NtSOD*-F**	5′-ATTACCGACAAGCAGATTCCTC-3′
**q*NtSOD*-R**	5′-CAACCCTTCCACCAGCATTTCC-3′
**q*NtCAT1*-F**	5′-TGGATCTCATACTGGTCTCA-3′
**q*NtCAT1*-R**	5′-TTCCATTGTTTCAGTCATTCA-3′
**q*NtERD10C*-F**	5′-CATGATGACCTGGCAACTTC-3′
**q*NtERD10C*-R**	5′-AGCTCTAGCTCGGAGGATGA-3′
**q*NtLEA5*-F**	5′-CTCTAACTCCAAACTCATCTCT-3′
qNtLEA5-R	5′-CAAAACCCCAGATTCAAGAC-3′
qNtDREB1A-F	5′-ACTGGACGTCCTGAGTGACA-3′
qNtDREB1A-R	5′-GGCATCGGAAGCCAGAAAAG-3′
qNtCOR15A-F	5′-GTCGTCGTTTCTCAACGCAAGA-3′
qNtCOR15A-R	5′-GCTTTCTCAGCTTCTTTACCCA-3′
qNtCOR47-F	5′-TGTCATCGAAAAGCTTCACCGA-3′
qNtCOR47-R	5′-ACCGGGATGGTAGTGGAAACTG-3′
qNtKIN1-F	5′-ATGCCTTCCAAGCCGGTCAGAC-3′
qNtKIN1-R	5′-CCGGTCTTGTCCTTCACGAAGT-3′
